# Editorial: Advanced monitoring in ARDS: enhancing mechanical ventilation through innovative techniques

**DOI:** 10.3389/fmed.2025.1756943

**Published:** 2026-01-09

**Authors:** Francesco Murgolo, Michele Bertoni, Savino Spadaro, Salvatore Grasso

**Affiliations:** 1 Section of Anesthesiology and Intensive Care Medicine, Department of Precision-Regenerative Medicine and Jonic Area (DiMePRe-J), University of Bari “Aldo Moro”, Bari, Italy; 2 Interdepartmental Division of Critical Care Medicine, Medical and Surgical Intensive Care Unit, Toronto General Research Institute, University Health Network, Toronto, ON, Canada; 3 Department of Medical and Surgical Specialties, Radiological Sciences and Public Health, University of Brescia, Brescia, Italy; 4 Department of Emergency, ASST Spedali Civili University Hospital, Piazzale Ospedali Civili, Brescia, Italy; 5 Section of Anesthesiology and Intensive Care Medicine, Department of Translational Medicine, University of Ferrara, Ferrara, Italy

**Keywords:** acute respiratory failure, ARDS, EIT, lung ultrasonography (LUS), mechanical ventilation

Optimizing mechanical ventilation in patients with acute respiratory distress syndrome (ARDS) remains a cornerstone of critical care. While the principles of lung-protective ventilation such as low tidal volume, limited plateau pressure, and adequate positive end-expiratory pressure (PEEP) are well established, lung injury is highly heterogeneous. Regions of collapse and overdistension often coexist, making global parameters insufficient; therefore, the development of advanced monitoring techniques has become essential to facilitate disease phenotyping and support tailored ventilatory management. In this Research Topic, we collect studies focused on bedside monitoring approaches that may help refine ventilatory strategies and potentially mitigate the risk of ventilator-induced lung injury (VILI).

Pettenuzzo et al. provide a comprehensive overview of current and emerging bedside tools for guiding lung-protective ventilation. Among these, electrical impedance tomography (EIT) stands out as a non-invasive, radiation-free technique capable of generating real-time regional images of lung aeration. Briefly, by tracking changes in thoracic electrical impedance throughout the respiratory cycle, EIT offers continuous insight into the distribution of ventilation, enabling clinicians to detect patterns of overdistension and collapse and, more broadly, the heterogeneous morphology that characterizes ARDS. This ability to capture regional dynamics at the bedside makes EIT particularly valuable for assessing the mechanical impact of ventilatory settings on the injured lung. The case report by Musaj et al., published in this series, represents a clear example of this potential: in a patient with severe unilateral lung injury, the authors performed near-daily EIT-guided PEEP titrations combined with esophageal pressure monitoring. This strategy allowed clinicians to adapt ventilation to evolving lung mechanics, progressively reducing PEEP from 18 to 8 cmH_2_O as recovery advanced, while minimizing both alveolar collapse and overdistension. This example clearly demonstrates how EIT can complement traditional imaging and support precision ventilation at the bedside. Yet, translating this potential into routine practice remains challenging, as device availability, the need for specialized expertise, and the absence of standardized protocols continue to limit its widespread adoption. Overcoming these barriers through training, protocol harmonization, and further clinical validation will be essential to fully realize the promise of EIT in guiding protective ventilation (1, 2).

In contrast, lung ultrasound (LUS) has already demonstrated its clinical utility as a simple, low-cost bedside tool (3, 4). Its portability and ease of use make it particularly attractive in resource-limited settings, where advanced technologies such as EIT may not be available. Beyond its established role in detecting pleural effusion and consolidations, recent studies have explored applications such as pleural strain assessment, which may provide additional insights into regional mechanics and open new perspectives for individualized ventilatory management. While LUS cannot provide the same continuous regional monitoring as EIT, its accessibility and growing evidence base position it as a practical and widely applicable complement to more sophisticated techniques.

The horizon of precision ventilation extends beyond imaging. As highlighted in their review, Pettenuzzo et al. explore research areas that are even more specialized and distant from routine clinical practice, such as the use of molecular markers and genetic signatures related to cellular stretch. Molecules like Clara Cell Secretory Protein-16 (CC-16) and specific microRNAs have been proposed as potential early indicators of ventilator-induced lung injury (VILI), paving the way for biologically guided ventilation. However, these approaches remain experimental and require robust validation before they can be integrated into clinical workflows.

Meanwhile, the enduring value of respiratory mechanics should not be underestimated. Simple bedside maneuvers, such as an end-inspiratory occlusion, allow measurement of plateau pressure and calculation of respiratory system compliance, parameters that are far from “mere numbers.” Indeed, respiratory system compliance reflects the amount of aerated lung available for ventilation, and driving pressure (tidal volume divided by compliance) serves as a practical tool to tailor tidal volume to the functional lung, reducing overdistension and cyclic strain (5–7). Moreover, combined with respiratory rate, these measurements enable estimation of mechanical power (the energy delivered to the lung parenchyma) helping clinicians strike the most efficient balance between tidal volume and frequency to minimize ventilatory energy load (8).

The integration of advanced monitoring tools and respiratory mechanics represents a significant step toward individualized ventilatory strategies in ARDS. While technological and logistical barriers persist, the trajectory is clear: precision medicine at the bedside is no longer a distant goal but an emerging reality. Bridging innovation with accessibility will be key to ensuring that these advances translate into improved outcomes for critically ill patients (9, 10).
